# Common and female-specific roles of protein tyrosine phosphatase receptors N and N2 in mice reproduction

**DOI:** 10.1038/s41598-023-27497-4

**Published:** 2023-01-07

**Authors:** Srdjan J. Sokanovic, Stephanie Constantin, Aloa Lamarca Dams, Yuta Mochimaru, Kosara Smiljanic, Ivana Bjelobaba, Rafael M. Prévide, Stanko S. Stojilkovic

**Affiliations:** 1 Section on Cellular Signaling, The Eunice Kennedy Shriver National Institute of Child Health and Human Development, National Institutes of Health, Bethesda, MD 20892 USA; 2 Department for Neurobiology, Institute for Biological Research “Siniša Stanković” - National Institute of Republic of Serbia, University of Belgrade, Bulevar Despota Stefana 142, 11000 Belgrade, Serbia

**Keywords:** Reproductive biology, Endocrinology

## Abstract

Simultaneous knockout of the neuroendocrine marker genes *Ptprn* and *Ptprn2*, which encode the protein tyrosine phosphatase receptors N and N2, causes infertility in female mice while males are fertile. To elucidate the mechanism of the sex-specific roles of *Ptprn* and *Ptprn2* in mouse reproduction, we analyzed the effects of their double knockout (DKO) on the hypothalamic-pituitary–gonadal axis. In DKO females, delayed puberty and lack of ovulation were observed, complemented by changes in ovarian gene expression and steroidogenesis. In contrast, testicular gene expression*,* steroidogenesis, and reproductive organs development were not significantly affected in DKO males. However, in both sexes, pituitary luteinizing hormone (LH) beta gene expression and LH levels were reduced, as well as follicle-stimulating hormone beta gene and gonadotropin-releasing hormone (GnRH) gene, while the calcium-mobilizing and LH secretory actions of GnRH were preserved. Hypothalamic *Gnrh1* and *Kiss1* gene expression was also reduced in DKO females and males. In parallel, a significant decrease in the density of immunoreactive GnRH and kisspeptin fibers was detected in the hypothalamic arcuate nucleus of DKO females and males. The female-specific kisspeptin immunoreactivity in the rostral periventricular region of the third ventricle was also reduced in DKO females, but not in DKO males. These data indicate a critical role of *Ptprn* and *Ptprn2* in kisspeptin-GnRH neuronal function and sexual dimorphism in the threshold levels of GnRH required to preserve reproductive functions.

## Introduction

Protein tyrosine phosphatase receptor types (PTPR) are a family of plasma membrane receptors composed of an extracellular domain that shares homology with cell adhesion molecules, one transmembrane domain, and one intracellular catalytic domain. Such a structure allows direct coupling of extracellular adhesion-mediated events with the regulation of intracellular signaling pathways^1^. Two proteins of this receptor family, PTPRN and PTPRN2, share such a structure, but do not show the tyrosine phosphatase activity^2,3^. However, their down- and up-regulation cause different outcomes, suggesting that they are operative in vivo^4^, but the mechanism of their action has not been clarified. PTPRNs are marker proteins of neuroendocrine cells^4,5^ and have partial homology with regulated endocrine-specific protein-18^6,7^, another marker protein of neuroendocrine cells^6,8^. These proteins appear to be present in the dense core secretory vesicles of neuroendocrine cells^9–11^. PTPRN has also been found in human glioma cells^12^ and PTPRN2 in metastatic breast cancer cells, where it is expressed in plasma membranes^13^. PTPRN2 has also been reported to cycle between the plasma membrane, vesicles, and the Golgi compartment^14^. Such localization of these proteins is in line with their proposed role in vesicle-mediated secretory processes^10,15,16^ and as promoters of cell migration and proliferation in metastatic tissues^12,13^.

One of the most interesting findings in this area is the sex-specific role of the PTPRN proteins in reproductive functions^16^. In female mice, knockout of both genes caused infertility, while most male double knockout (DKO) mice were fertile. In parallel, luteinizing hormone (LH) levels appeared to be reduced in the pituitary gland in females but were within the normal limits in males. Histological analysis of ovaries revealed antral follicles of normal appearance, but absence of corpus luteum. Treatment of DKO females with gonadotropins restored corpus luteum formation. These studies led the authors to conclude that knocking out *Ptprn* and *Ptprn2* alters the structure and function of dense core secretory vesicles, leading to reduced LH release and infertility in female mice^16^. However, no explanation was given as to how the fusion of LH-containing dense core secretory vesicles was affected in female but not male gonadotrophs. In pancreatic beta cells, which also express *Ptprn* and *Ptprn2*^9,11^, regulated exocytosis is directly linked to insulin expression via the PTPRN cytosolic fragment^17^. Further studies by the same group identified STAT5 as a binding domain for the PTPRN cytosolic fragment^18^ and the contribution of cyclin D in this process^19^.

To address the sexually dimorphic influence of PTPRN and PTPRN2 on reproduction, we used the same animal models^16^ and analyzed gene and hormone status in the hypothalamic-pituitary–gonadal axes of male and female mice. Our initial aim was to elucidate whether depletion of the LH secretory pool in DKO animals is sex-specific and whether LH release is reduced in DKO females but not in males. Furthermore, we wanted to elucidate the relationship between the LH beta gene (*Lhb*) and intrapituitary LH cell content in both females and males. We also examined whether the basal and gonadotropin-releasing hormone (GnRH) agonist stimulated LH secretion in vitro and in vivo is affected in a sex-specific manner and whether impaired LH release reflects inhibition of calcium signaling in female gonadotrophs. We further analyzed the expression of *Ptprn* and *Ptprn2* in hypothalamic, pituitary, and gonadal tissues and the status of GnRH (*Gnrh1*) and the kisspeptin (*Kiss1*) gene and protein expression in the hypothalamus and gonad-specific gene expression and steroid hormone production.

## Results

### Sex-specific effects of Ptprn^-/-^ + Ptprn2^-/-^ knockout on reproduction

Experiments were performed using the C57/Bl6 female and male mice: wild type (WT), single knockout *Ptprn*^*-/-*^ and *Ptprn2*^*-/-*^, and double (*Ptprn*^*-/-*^ + *Ptprn2*^*-/-*^*)* knockout (hereafter DKO), which were generated previously as described^20^. Consistent with initial report^16^, in our experimental conditions female and male single knockouts showed no effect on fertility and litter size, as well as *Ptprn*^*-/-*^ + *Ptprn2*^+*/-*^ couples, which were used as parents to generate DKO animals. In further agreement with previous work^16^, DKO females were infertile. The fertility of 90% of DKO males was like that of WT males, while about 10% of DKO males showed reduced fertility in relation to the number of pregnancies over time; however, the number of pups per litter was similar. Supplementary Fig. S1 illustrates that *Ptprn* and *Ptprn2* are expressed in the pituitary gland and hypothalamus, but not in gonads of WT females and males. Our single cell RNA sequencing of rat pituitary cells shows that *Ptprn* and *Ptprn2* are well expressed in both female and male gonadotrophs and all other hormone-producing cell types, as well as in folliculostellate cells of both sexes (NCBI Gene Expression Omnibus, GSE: 132,224). Therefore, sex-specific changes in hypothalamic and/or pituitary functions account for the observed changes in the reproductive status of DKO animals.

**Figure 1:**
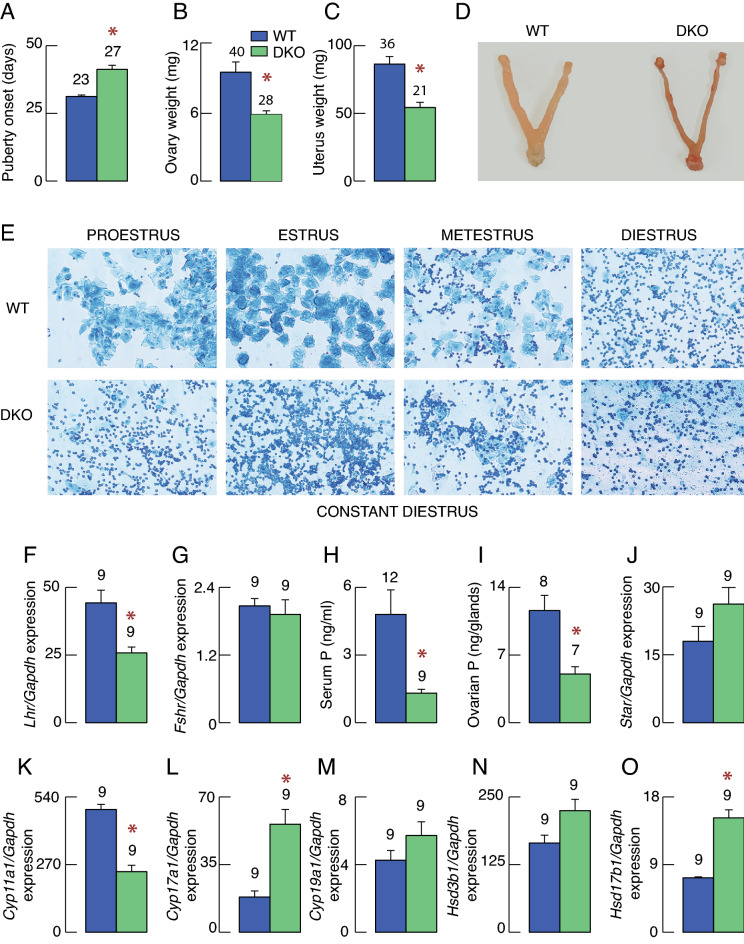
Effects of *Ptprn* and *Ptprn2* double knockout (DKO) on the female reproductive system. (**A**) Difference in onset of puberty in wild type (WT) and DKO females. (**B**–**D**) Reproductive organs in WT females in the diestrus stage of the estrous cycle and DKO females: the averaged weight of ovaries (B) and uterus (C) and representative examples of these organs (D). (**E**) Representative vaginal smears in WT and DKO females. Smears were taken daily for two weeks. (**F**,**G**) Expression of gonadotropin hormone receptor genes *Lhr* (F) and *Fshr* (G) in ovaries. (**H,I**) Gonadal steroid hormones in WT females in the diestrus stage of the estrous cycle and DKO females: serum (H) and ovarian (I) progesterone. (**J**–**O**) Ovarian steroidogenic gene expression in WT and DKO animals: *Star* (J), *Cyp11a* (K), *Cyp17a1* (L), *Cyp19a1* (M), *Hsd3b1* (N) and *Hsd17b1* (O). In this and following figures, bars shown are mean ± SEM values; dark blue bars denote WT and green bars DKO animals; numbers above bars indicate number of replicates per group; asterisks, P < 0.01 between pairs. The age of animals (days) was WT = 110 ± 3.6 (N = 40); DKO = 115 ± 3.8 (N = 28); animal body weight (g) was WT = 20.65 ± 0.28; DKO 21.32 ± 0.50.

Here we focus on elucidating elements of the hypothalamic-pituitary–gonadal axis that are sensitive to loss of *Ptprn* and *Ptprn2* expression. DKO females showed a significant delay in vaginal opening compared to controls (Fig. 1A). At around 100 days of postnatal age, ovaries (Fig. 1B) and uteri (Fig. 1C) from DKO females showed significantly lower weights when compared with WT females in diestrus stage of estrous cycle. A representative example of these organs is shown in Fig. 1D. In contrast to WT females exhibiting a 4–5-day estrous cycle (Fig. 1E, top), DKO females were in constant diestrus (Fig. 1E, bottom), indicating the lack of ovulation. The expression of LH receptor gene (*Lhr*), which controls gonadal steroidogenesis^21^, was significantly reduced (Fig. 1F), but not the expression of follicle-stimulating hormone (FSH) receptor gene (*Fshr*) controlling gametogenesis (Fig. 1G)^22^.

Serum progesterone levels (Fig. 1H) were also significantly lower in DKO females as were progesterone levels in ovarian tissues (Fig. 1I) compared to WT females in the diestrus stage of the estrous cycle. The expression of *Star* gene, encoding steroidogenic acute regulatory protein that plays a key role in the rapid transport of cholesterol from the outer to the inner mitochondrial membrane^23^, was not affected (Fig. 1J). However, we observed a significant decrease in cytochrome P450 family 11 subfamily A member 1 gene (*Cyp11a1*) expression (Fig. 1K), which encodes CYP11A1 enzyme that catalyzes cleavage of cholesterol to pregnenolone, the first and rate limiting step in steroid biosynthesis^24^. The expression of genes controlling further steps in gonadal steroidogenesis were either stimulated (cytochrome P450 family 17 subfamily A member 1—*Cyp17a1* and hydroxysteroid 17-beta dehydrogenase 1—*Hsd17b1*) or not affected (cytochrome P450 family 19 subfamily A member 1—*Cyp19a1* and hydroxy-delta-5-steroid dehydrogenase, 3 beta- and steroid delta-isomerase 1—*Hsd3b1*) (Fig. 1L–O).

**Figure 2:**
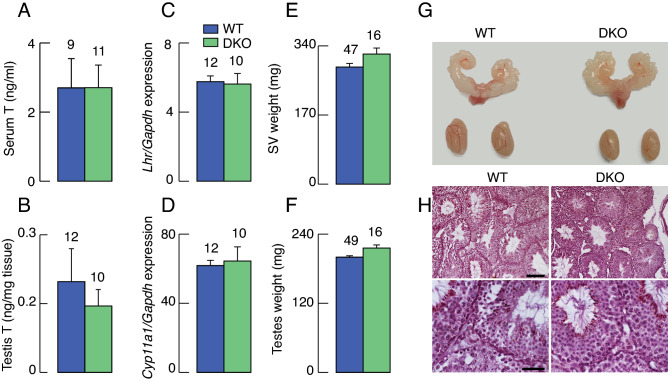
Lack of effects of DKO on male reproductive system. (**A**,**B**) Testosterone levels in WT and DKO animals: serum (A) and testes (B). (**C**,**D**) Expression of testicular genes in WT and DKO animals: *Lhr* (C) and *Cyp11a1* (D); the expression of other testicular genes was also comparable in WT and DKO animals, including *Fshr, Cyp17a1*, *Hsd17b1*, and *Hsd3b1.* **(E–H)** Reproductive organs in WT and DKO males: mean ± SEM weight values for seminal vesicles (E), testes (F), representative examples of these organs (G), and histological evidence for normal spermatogenesis and spermiogenesis in both groups using hematoxylin staining; scale bars: 100 µm for upper and 40 µm for lower panel (H). Data shown are mean ± SEM values; with numbers of replicates per group shown above bars; asterisks, P < 0.01 between pairs. The age of the animals (days) was WT = 103.24 ± 1.66 (N = 49); DKO = 108.9 ± 3.08 (N = 16). The body weight (g) of the animals was WT = 27.85 ± 0.31; DKO = 27.56 ± 0.47.

In contrast, DKO male mice had normal serum (Fig. 2A) and testicular (Fig. 2B) testosterone levels and comparable expression of *Lhr* (Fig. 2C) and *Cyp11a1* (Fig. 2D) in testicular tissue. The expression of other testicular genes was also not affected: *Fshr* (WT = 0.60 ± 0.03, DKO = 0.67 ± 0.03); *Star* (WT = 12.77 ± 0.76, DKO = 12.70 ± 1.93); *Cyp17a1* (WT = 56.52 ± 2.33, DKO = 74.97 ± 10.99); and *Hsd3b1* (WT = 15.43 ± 1.44, DKO = 11.85 ± 0.97); values derived from N = 12 for WT, and N = 10 for DKO. In accordance with normal testicular steroidogenesis, development of seminal vesicles and testes in DKO mice was comparable to the WT animals (Fig. 2E–G) and histological analysis confirmed spermatogenesis and spermiogenesis, with spermatozoa present in tubule lumen of both WT and DKO animals (Fig. 2H).

### DKO inhibits *Lhb* expression and LH accumulation in pituitary glands

**Figure 3:**
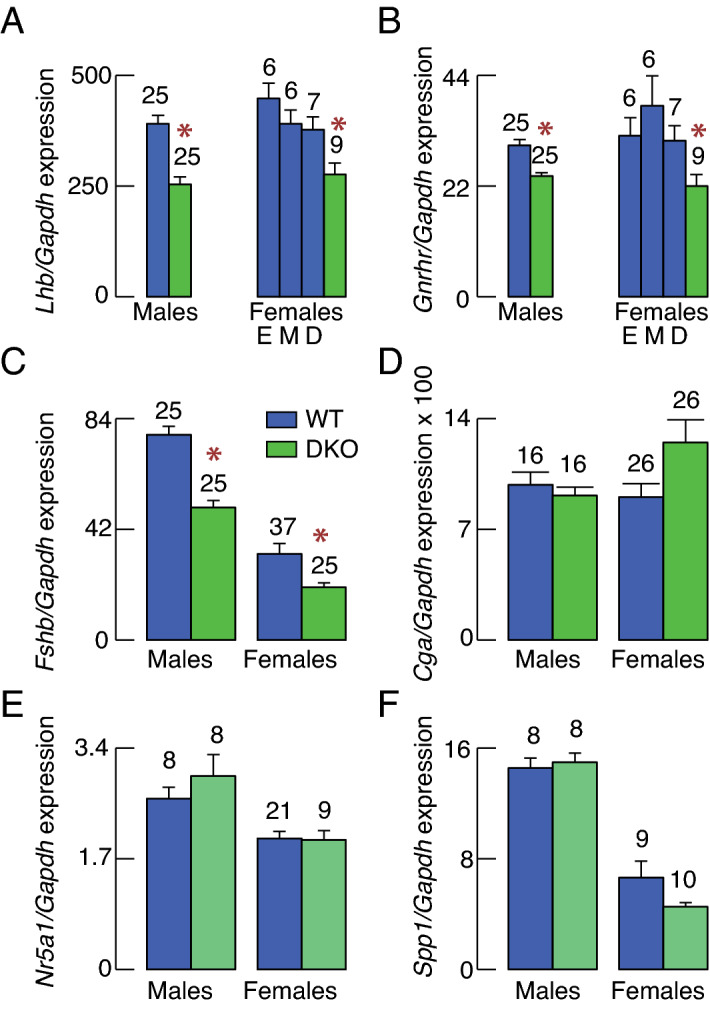
Selective effects of DKO on gonadotroph-specific gene expression. (**A**–**F**) Gene expression in male and female WT and DKO mice: *Lhb* (A), *Gnrhr* (B), *Fshb* (C), *Cga* (D), *Nr5a1* (E), and *Spp1* (F). Results for WT females were derived from animals in estrus (E), metestrus (M) and diestrus (D) stage of estrous cycle (A and B), or from random cycling animals (C–F). The data shown are mean ± SEM values and the numbers of replicates are indicated above the bars. Asterisks, P < 0.01 between DKO and each control group (A and B) or between pairs (C).

Within pituitary cell types, gonadotrophs specifically express several genes, including *Lhb, Fshb, Gnrhr,* nuclear receptor subfamily 5 group A member 1 (*Nr5a1*), and secreted phosphoprotein 1 (*Spp1*) and together with thyrotrophs also express glycoprotein hormones, alpha polypeptide gene (*Cga*)^8,25^. Here we analyzed the expression of these genes in pituitary glands from WT and DKO females and males (Fig. 3). The expression of *Lhb* was significantly reduced in pituitaries of DKO females compared to WT females in estrus, metestrus and diestrus, with percentage reduction of 40%, 38% and 28%, respectively. *Lhb* expression was also significantly reduced in DKO males by 36% (Fig. 3A). Similarly, *Gnrhr* expression was significantly reduced in DKO females compared to estrous, metestrus, and diestrus controls by 31%, 42%, and 29%, and in DKO males for 20% (Fig. 3B). *Fshb* expression was also significantly reduced in DKO females and males, compared with random cycling females (by 37%) and control males (by 35%) (Fig. 3C). In contrast, no significant changes in expression of *Cga* was observed in DKO animals, when compared to WT animals (Fig. 3D). The expression of *Nr5a1* (Fig. 3E) and *Spp1* (Fig. 3F) was also not affected in DKO males and females. These results indicate that there is no sex-specific difference in gonadotroph-specific gene expression in DKO animals compared to age-matched controls. Transcription of *Lhb, Fshb*, and *Gnrhr* is stimulated by GnRH^26^ and *Spp1* expression is unaffected by GnRH^27^, suggesting that endogenous GnRH secretion is reduced in DKO animals.

**Figure 4:**
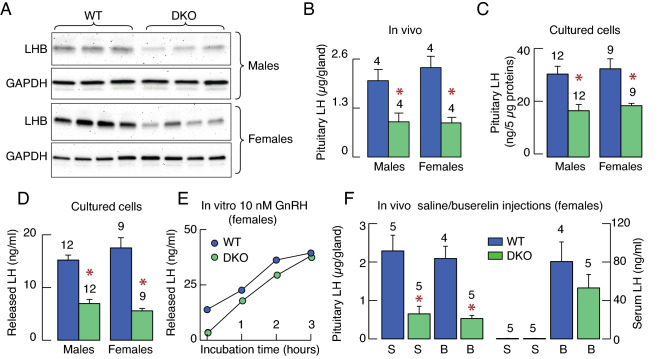
Decreased LH levels in the pituitary gland of DKO animals limit LH secretion. (**A**) Western blot analysis of LHB of male (top panel) and female (bottom panel) pituitaries in WT and DKO mice. The data shown are representative from five experiments. The blots presented were cropped from the original immunoblots, which are shown in Supplementary Figs. S2–S5. (**B**,**C**) LH content in pituitary tissue (B) and cultured pituitary cells (C) from WT and DKO mice assessed by ELISA. (**D**) Basal LH release by cultured pituitary cells during 4 h incubation. (**E**) Time course of GnRH-stimulated LH release in cultured pituitary cells. (**F**) Pituitary LH content (left panel) and serum LH concentration (right panel) 90 min after the intraperitoneal injection of saline (S) or 0.5 µg buserelin acetate (B), a GnRH receptor agonist, in WT and DKO mice. The bars shown are mean ± SEM values; number of replicates are indicated above the bars; asterisks, P < 0.01 between pairs. The circles indicate mean values from duplicate determination. Dissociated pituitary cells (120,000/well) were cultured for two days prior to experiment.

In further experiments, we examined whether the reduction in *Lhb* expression affects the synthesis and release of LH. In the first experiment, we used western blotting to analyze LHB content in male (Fig. 4A, top) and female (Fig. 4A, bottom) pituitaries from WT in the diestrus stage of estrous cycle and DKO mice. Contrary to the sex-specific effects of DKO on reproduction, we observed lower density of LHB bands in both DKOs, females and males, when compared to the WT animals of the same age. In females, we further compared the density of LHB bands from DKO females with bands from WT animals in proestrus, estrus, and diestrus stage of estrous cycle. This analysis confirmed that density of LHB bands was lower in DKO females than in any stage of estrous cycle in WT females (proestrus = 1 ± 0.12, DKO = 0.36 ± 0.06; estrus = 1 ± 0.08, DKO = 0.52 ± 0.09; diestrus = 1 ± 0.08, DKO = 0.51 ± 0.11).

We also measured LH cell content in pituitaries from WT and DKO males and females using ELISA assay. This analysis confirmed a significant decrease in LH cell content per pituitary from DKO females and males when compared to WT animals of the same age (Fig. 4B). In third experiment, we dissociated cells from WT and DKO pituitaries and cultured them for 2 days, followed by examination of intracellular LH content and basal release of this hormone during 4 h incubation. As shown in Fig. 4C, pituitary cells from DKO animals still showed lower intracellular LH concentration two days after dissociation. To examine effects of DKO on basal (in the absence of GnRH) LH release, a fraction of these cells from females was incubated in a shaking water bath for 4 h at 37 °C. At the end of incubation, media were collected, and LH was measured by ELISA. In both cell cultures, from females and males, basal LH release was significantly lower in cells from DKO animals (Fig. 4D). Therefore, it is reasonable to conclude that DKO-dependent reduction of *Lhb* expression affects LH synthesis in pituitary gonadotrophs from females and males.

We also examined the in vitro and vivo effects of GnRH on LH secretory responses to clarify whether the LH exocytotic pathway was inhibited in DKO females, as suggested earlier^16^. Addition of 10 nM GnRH to cultured pituitary cells from females stimulated LH secretion in a time-dependent manner, with comparable amplitude of responses in cultures from WT and DKO pituitaries (Fig. 4E). For in vivo evaluation, female mice were *ip* injected with 0.5 µg buserelin acetate, a GnRH receptor agonist, and were euthanized 90 min after injection, blood was collected, and pituitaries were removed to measure LH by ELISA. Pituitary LH content was comparable in saline and buserelin-treated WT animals and significantly lower in DKO animals (Fig. 4F, left). However, in both WT and DKO animals, serum LH levels were significantly elevated in buserelin-treated animals when compared to saline-treated animals (Fig. 4F, right). These results challenge the original hypothesis that the exocytotic pathway is inhibited in the absence of *Ptprn* and *Ptprn2*^16^.

**Figure 5:**
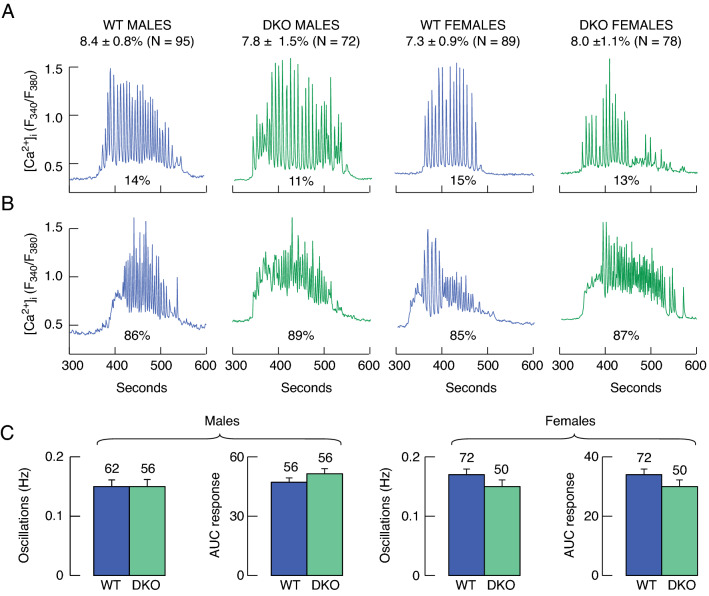
GnRH (100 pM) induced calcium signaling in cultured pituitary cells. Dissociated pituitary cells were cultured for two days, washed, and calcium recording was done in Fura-2 loaded single cells during the first 5 min in the absence of GnRH (not shown), followed by 3 min application of 100 pM GnRH and washout of GnRH for 2 min. Experiments were performed with cells derived from WT and DKO male and female mice. The averaged percentage of responding cells is shown above traces as mean ± SEM values. N indicate total number of cells responding to GnRH with the threshold calcium signaling. (**A**) A smaller fraction of cells responded to GnRH with more regular calcium oscillations. (**B**) The majority of cells responded with damped oscillations. (**C**) Quantification of intracellular calcium response to GnRH in gonadotrophs from WT and DKO males and females: frequency of spiking and the area under the curve (AUC) of the calcium response.

Finally, we studied GnRH-induced calcium signaling in gonadotrophs from WT and DKO males and females to elucidate whether GnRH receptor efficacy is lower due to reduced *Gnrhr* expression. These experiments revealed that GnRH (100 pM) triggered calcium signaling in 7–8% of cells (Fig. 5, top row of numbers), which is largely consistent with the literature data on the percentage of gonadotrophs in the pituitary gland^28^, indicating that the drop in *Lhb* expression and intrapituitary LH content does not reflect the partial loss of gonadotrophs in the anterior pituitary. There are three types of GnRH-induced calcium signals, depending on its concentrations and response patterns: baseline oscillations, when calcium concentrations fall to initial concentrations with each spike (low-dose GnRH), "dampened" oscillations, when spikes do not reach baseline (intermediate doses), and biphasic non-oscillatory response (high doses)^29^. In cultured pituitary cells 334 cells from four cell preparations responded to 100 pM GnRH with oscillatory calcium signaling: WT males, DKO males, WT females, and DKO females (Fig. 5, top). A minority of cells responded to GnRH application with regular oscillatory calcium signaling (Fig. 5A) and the most gonadotrophs responded with dampened oscillations (Fig. 5B). There were no significant differences in the frequency of calcium spiking and area under the curve (AUC), with the later number reflecting the amplitude of the response and pattern of calcium signaling (Fig. 5C). Overall, these results are consistent with a decrease in *Lhb* expression in DKO gonadotrophs, suggesting that LH synthesis is inhibited in DKO animals rather than GnRH-induced calcium signaling and/or calcium-regulated LH exocytosis.

### Hypothalamic GnRH and kisspeptin are reduced in DKO animals

**Figure 6:**
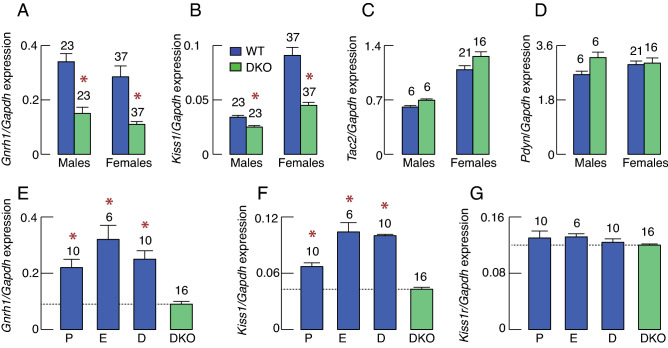
Selective effects of DKO on gene expression in the hypothalamus. (**A**–**D**) Gene expression in neurons that contribute to GnRH pulse generator*: Gnrh1* (A), *Kiss1* (B), *Tac2* (C), and *Pdyn* (D). (**E**–**G**) Comparison of gene expression in WT females at different stages of the estrous cycle and DKO females: *Gnrh1* (E), *Kiss1* (F), and *Kiss1r* (G). Bars shown are mean ± SEM values; numbers above bars indicate number of replicates; asterisks, P < 0.01 between pairs or between DKO and control proestrus (P), estrus (E), and diestrus (D).

The dependence of pituitary gonadotroph function on hypothalamic GnRH is well established^30^ and may provide a rationale for decreased *Lhb* expression and LH synthesis in DKO females and males. To address this issue, in further studies we analyzed the expression of *Gnrh1, Kiss1*, *Tac2*, and *Pdyn* genes in hypothalamic tissues, which neurons contribute to the formation of a network accounting for pulsatile and surge modes of GnRH release^31^. These experiments showed a significant decrease of *Gnrh1 e*xpression by 56% and 60% (Fig. 6A) and *Kiss1* expression by 28% and 50% (Fig. 6B) in DKO males and females, respectively. In contrast, the expression of *Tac2* (Fig. 6C) and *Pdyn* (Fig. 6D) was comparable in hypothalamic tissues from WT and DKO females and males. We also analyzed the expression of *Gnrh1*, *Kiss1*, and *Kiss1r* in different stages of estrous cycle. Results indicated that *Gnrh1* (Fig. 6E) and *Kiss1* (Fig. 6F) expression was significantly lower in DKO females than in any stage of estrous cycle of WT animals. Interestingly, the expression of *Kiss1r* was not affected in DKO females (Fig. 6G). Therefore, it is reasonable to conclude that decreased GnRH synthesis reflects down-regulation of *Gnrh1* and *Kiss1* gene expression in DKO males and females, causing a decrease in *Lhb* expression and LHB synthesis in pituitary gonadotrophs.

**Figure 7:**
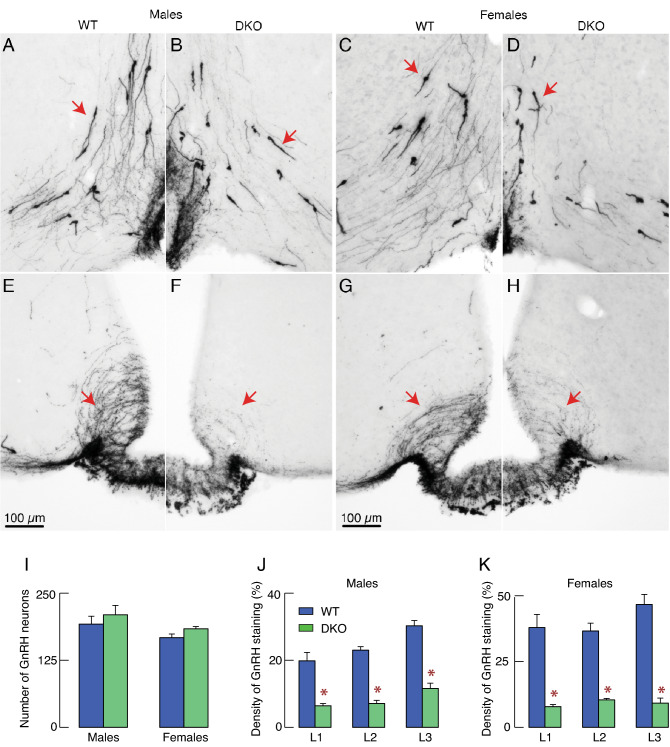
Effects of DKO on the distribution and functions of GnRH-secreting hypothalamic neurons. (**A**–**D**) GnRH cell body distribution in the hypothalamus. Red arrows, representative examples of GnRH cell bodies. Pattern of immunopositive GnRH neuron expression in WT (A) and DKO males (B) and WT (C) and DKO females (D). **(E**–**H**) GnRH fibers through the arcuate nuclei; red arrows, representative examples. GnRH immunoreactivity in WT (E) and DKO males (F) and WT (G) and DKO females (H). Horizontal bars of 100 µm also apply to all panels. (**I**–**K**) Quantification of the effect of DKO on the hypothalamic GnRH-secreting neurons. (**I**) Number of GnRH neurons per brain series (i.e. 25% of the brain). (**J**,**K**) Density of GnRH staining through the arcuate nucleus of males (J) and females (K). L1–L3, different levels of the arcuate nuclei, as described in Material and Methods. Data shown are mean ± SEM from six measurements per region; asterisks, P < 0.01 between pairs.

We also analyzed the cell distribution and number of hypothalamic GnRH neurons WT and DKO females and males using immunohistochemistry. DKO did not alter GnRH cell body distribution nor had a visible effect on GnRH cell body number in males (Fig. 7A,B) and females (Fig. 7C,D; representative neurons are indicated by arrows). However, there was an apparent reduction in GnRH immunoreactivity throughout the arcuate nucleus that GnRH fibers cross to reach the median eminence (indicated by arrows in Fig. 7E–H) in DKO animals compared to WT males (Fig. 7E,F) and WT females (Fig. 7G,H). In contrast, the GnRH fiber density projecting to organum vasculosum laminae terminalis did not appear different between DKOs and WTs (Fig. 7A–D). Quantification analysis confirmed no significant changes in the number of GnRH neurons in DKO females and males (Fig. 7I) but a significant decrease of GnRH fiber density in the arcuate nuclei of males (Fig. 7J) and females (Fig. 7K) DKOs were detected. These results suggest that the decrease in *Gnrh1* expression and/or GnRH secretion might account for decrease in *Lhb* expression and LH synthesis in both females and males.

**Figure 8:**
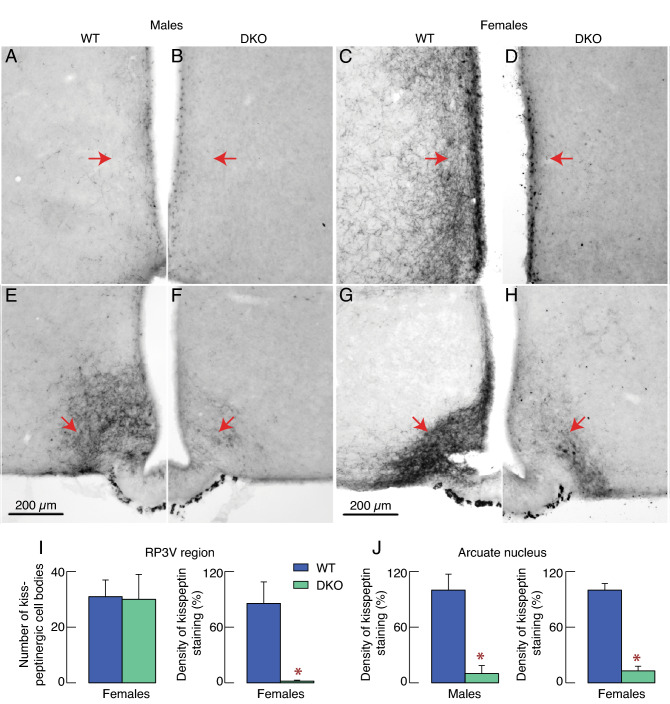
Common and female-specific effects of DKO on the hypothalamic kisspeptin-secreting neurons. (**A–D**) Kisspeptin immunoreactivity in the rostral periventricular region of the third ventricle. Pattern of kisspeptin immunoreactivity of WT (A) and DKO males (B) and WT (C) and DKO females (D). (**E**–**H**) Kisspeptin immunoreactivity in the arcuate nucleus. Pattern of kisspeptin immunoreactivity of WT (E) and DKO males (F) and WT (G) and DKO females (H). Horizontal bars of 200 μm apply to all panels. Arrows indicate representative fiber density in the RP3V (A – D) and the arcuate nucleus (E–H). (**I**) Quantification of kisspeptinergic cell bodies (left panel) and fiber densities (right panel) in the RP3V region of WT and DKO females whose representative matched sections are shown in C-D. (**J**) Quantification of kisspeptinergic fiber densities in the arcuate nucleus of WT and DKO males (left panel) and females (right panel) whose representative matched sections are shown in E–F and G–H, respectively.

The kisspeptin immunoreactivity in hypothalamic tissues of WT and DKO animals was also analyzed. As indicated by red arrows in Fig. 8, kisspeptin immunoreactivity was detected in the rostral periventricular region of the third ventricle (RP3V) (Fig. 8A–D) and the arcuate nucleus (Fig. 8E–H). As expected, moderate immunoreactivity in RP3V was detected in both WT (Fig. 8A) and DKO male animals (Fig. 8B). However, immunoreactive kisspeptin fibers were dense in the RP3V of WT female animals (Fig. 8C) but undetectable in DKO animals despite that kisspeptinergic cell bodies were visible in DKO animals (Fig. 8D). Immunoreactive kisspeptin fibers were dense in the arcuate nucleus of WT males and females (Fig. 8E,G), but barely discernable in the arcuate nucleus of DKO males and females (Fig. 8F,H). Quantification of kisspeptinergic cell bodies in the RP3V region of WT and DKO females revealed no significant differences (Fig. 8I, left), but there was a statistically significant difference in fiber density (Fig. 8I, right). Quantification of kisspeptinergic fiber densities in the arcuate nucleus of WT and DKO males (Fig. 8J, left) and females (Fig. 8J, right) confirmed the presence of statistically significant differences in mean values between WT and DKO animals. These experiments demonstrated that hypothalamic changes, particularly in RP3V, may explain the sex-specific impairment of fertility due to its critical role in the onset of puberty and ovulation.

## Discussion

The focus of this study is on the sex-dimorphic effects of deletions of the *Ptprn* + *Ptprn2* genes on reproductive functions, previously reported by others^16^ and confirmed in our study; the majority of DKO females were infertile, while DKO males were fertile when mating with WT females. Their histological analysis revealed that spermatogenesis was preserved, as well as oogenesis, but with absence of corpus luteum and that treatment of DKO females with gonadotropins restored corpus luteum formation^16^. We also show here that in the ovaries, but not in the testes, of DKO animals, significant changes in steroidogenesis and gene expression, cause a delay in puberty and uterine development, as well as the absence of ovulation in DKO females. In female mice, knockdown of *Ptprn2* alone also delays the onset of puberty^32^.

The sex-specific effects of DKO may be related to the loss of functions of these two genes in the gonads, pituitary gland, and/or hypothalamus. Our experiments clearly show the lack of expression of these genes in ovary and testis, which is consistent with the PTPRN and PTPRN2 protein expression in human pituitary and hypothalamus, but not in gonads^33^ (Human Protein Atlas proteinatlas.org). Using qRT-PCR analysis, we also showed the expression of these genes in pituitary tissue. Moreover, our recent single cell RNA sequencing of freshly dispersed rat pituitary cells revealed that *Ptprn* and *Ptprn2* are well expressed in both female and male gonadotrophs, as well as other types of pituitary secretory cells and folliculostellate cells of both sexes^8,25^. Kubosaki et al.^16^ also reported that these genes are expressed in the pituitary gland, as shown by immunohistochemistry. We have also shown that hypothalamic tissue express *Ptprn* and *Ptprn2*, and other reported that GnRH—and Kiss1-secreting neurons express these genes^34,35^. Thus, these data confirmed that *Ptprn* and *Ptprn2* are common neuroendocrine marker genes, and loss of their function in the pituitary and/or in hypothalamus accounts for a loss of female fertility.

Kubosaki et al.^16^ suggested that deletion of these genes affects pituitary gonadotroph functions, specifically that LH levels in the pituitary gland and serum are reduced in females, but not in males, leading to a lack of LH surge and ovulation. PTPRN + PTPRN2 proteins have been described as crucial for the structure and function of the dense core secretory vesicles^9,11^, which could imply sexual dimorphism in exocytotic LH release. If these conclusions are correct, they raised at least two important points: the sex specificity of *Ptprn* and *Ptprn2* expression in pituitary gonadotrophs and the nature of the specific role of these genes in the LH exocytotic pathways. Our scRNAseq ruled out the first hypothesis because the *Ptprn* and *Ptprn2* genes were observed in both female and male gonadotrophs^8^. Here, we also showed no difference in the rate of GnRH-induced LH release in WT and DKO females and cultured cells from these animals. Consequently, it is justified to conclude that the sexually dimorphic effects of the deletions of these genes on reproductive functions have been confirmed, but the accompanying hypotheses about the mechanism of this action should be rejected.

To understand the pathways that determine this sex-specific response to gene deletion, we first analyzed the effect of DKO on the expression of gonadotroph-specific genes, which play a key role in reproduction. This analysis revealed that *Lhb* expression was reduced in DKO females and males. Western blot analysis and ELISA measurements of pituitary LH in vivo and cultured cells from DKO animals also showed significantly lower LH levels not only in female DKO animals but also in male DKO mice compared to WT animals. Therefore, the level of reduction in *Lhb* expression in DKO females and males was sufficient to affect LH synthesis in pituitary gonadotrophs.

These results would be consistent with the hypothesis that deletion of *Ptprn* + *Ptprn2* genes in pituitary gonadotrophs directly causes a decrease in *Lhb* expression, leading to a decrease in LH synthesis and release. In parallel, it has been shown that regulated exocytosis of secretory vesicles in pancreatic beta cells is accompanied by cleavage of PTPRN at the plasma membrane in a calcium-dependent manner, generating a cytosolic fragment of this molecule that promotes insulin gene expression^17^. However, expression of *Fshb* and *Gnrhr* in gonadotrophs and *Gnrh1* and *Kiss1* in the hypothalamus was also reduced in DKO females and males, raising the question of whether PTPRNs are involved in the transcription of all these genes, which requires further investigation.

There are two other alternative hypotheses that need to be addressed. First, the finding that *Gnrhr* expression was also reduced in DKO animals raised the question of the expression and efficacy of GnRH receptors to drive calcium signaling and LH secretion, which could also provide a rationale for the reduced LH response. Single cell calcium recordings revealed similar patterns of GnRH-induced calcium signaling in male and female gonadotrophs obtained from WT and DKO mice. In response to 100 pM GnRH application, all gonadotrophs responded with oscillatory calcium signaling ranging from baseline oscillations to damped oscillations. Thus, although *Gnrhr* expression is significantly reduced in DKO animals, the amplitude of the reduction is unlikely to be sufficient to affect the GnRH-stimulated calcium signaling pathway and calcium-controlled exocytosis.

Second, it is well established that the expression of *Lhb, Fshb,* and *Gnrhr* in gonadotrophs is controlled by hypothalamic GnRH^36,37^, indicating indirect effects of DKO on LH synthesis, through changes in GnRH secretion. Consistent with this hypothesis, GnRH neurons are likely to express PTPRN and PTPRN2 proteins^34^. We also observed significant inhibition of *Gnrh1* expression in the hypothalamic tissues of DKO female and male mice. Reduced expression of *Gnrh1* did not alter the appearance of GnRH neurons in the hypothalamus, as assessed by neuronal distribution and cell body number. In contrast, there was a significant decrease in the density of GnRH staining in the arcuate nucleus in females and males, indicating that *Gnrh1* expression, GnRH processing, and/or trafficking were affected in DKO female and male mice, causing a decrease in *Lhb* expression and LH synthesis. However, these observations might not solely provide an explanation for the sexually dimorphic effects of DKO on reproduction.

Unlike males whose reproductive capacity relies on a pulsatile GnRH secretory pattern for spermatogenesis and steroidogenesis, female fertility relies on two GnRH secretory patterns: GnRH pulses for follicle maturation and steroidogenesis, and GnRH surge for ovulation^31^. Pulsatile GnRH/LH release is driven by a neuronal assemble termed “GnRH pulse generator” located in the arcuate nucleus^38^, with kisspeptin-secreting neurons playing a major role in its operation by regulating distal projections of GnRH neurons near the median eminence without affecting GnRH neuron cell bodies^39,40^. A hallmark of this subpopulation of arcuate kisspeptinergic neurons is the coexpression of two other neuropeptides, neurokinin B and dynorphin A, from which autoregenerative activity arises^41,42^. In female rodents, a distinct population of kisspeptin neurons is located in RP3V and stimulates the cell bodies of GnRH neurons to release GnRH, which accounts for the LH surge^31^. The pulsatile GnRH secretory pattern relies upon a minimum of ~ 20% of arcuate kisspeptinergic neurons^43^ and 12% of GnRH neurons^44^. However, the GnRH surge requires RP3V kisspeptin neurons and 12–34% of GnRH neurons^44^.

Here, we show that the expression of *Kiss1*, the gene encoding kisspeptin, is significantly reduced in the hypothalamus in DKO females and males, whereas the expression of *Tac2*, encoding tachykinin 2/neurokinin B, and *Pdyn*, encoding prodynorphin, were unaffected. These observations suggest that the function of kisspeptin neurons depends on the *Ptprn* + *Ptprn2* genes, which were detected in these cells^35^. Since dissection of the hypothalamus to perform qRT-PCR did not allow us to distinguish the two kisspeptinergic populations, we used immunohistochemistry to obtain the necessary spatial resolution. Consistent with the literature, kisspeptin immunoreactivity was dense in both the RP3V and the arcuate nucleus of WT females, as well as in the arcuate nucleus of WT males. Kisspeptin immunoreactivity was also detected in DKO animals but was reduced. In DKO females and males, arcuate nucleus kisspeptin fiber immunoreactivity was reduced to similar ranges in both sexes, but was still detectable. In DKO females, RP3V contained kisspeptinergic cell bodies but was devoid of kisspeptinergic fibers. This dichotomy may be supported by publicly available gene expression databases, which indicate that GnRH neurons and kisspeptin neurons from the arcuate nucleus express *Ptprn* + *Ptprn2* genes at low levels^34,45^, whereas kisspeptin neurons from the RP3V express these genes at high levels^35^.

These observations may also provide a rationale for the sexually dimorphic effects of the *Ptprn* + *Ptprn2* gene deletion on reproductive functions. Loss of kisspeptinergic fibers staining in RP3V would be consistent with lack of ovulation, as RP3V kisspeptin neurons express estrogen receptor alpha and transmit positive estradiol feedback to GnRH cell bodies to induce a GnRH surge at the median eminence^46,47^. RP3V kisspeptin neurons also transmit circadian signals from the suprachiasmatic nucleus to synchronize ovulation with the onset of the active period^48^. Reduction of kisspeptinergic fibers in the arcuate nucleus, combined with reduction of GnRH fibers, can also alter GnRH pulse amplitude, and explain reduced LH synthesis in both males and females. Delayed puberty in DKO females would also be expected due to the lack of connectivity between RP3V kisspeptin neurons and GnRH neurons. Indeed, increased RP3V kisspeptin neurons and kisspeptinergic inputs to cell bodies of GnRH neurons are hallmarks of peripubertal maturation in female rodents^49^ and fewer RP3V kisspeptin neurons are associated with delayed puberty^50^.

Therefore, contrary to what was previously concluded, the hypothalamus appears to be the primary component of the reproductive axis affected by DKO. While only two major populations of hypothalamic neuron have been examined, and other cannot be ruled out, these central defects are sufficient to establish a hypothesis explaining female infertility. Reduced *Kiss1* expression and kisspeptin synthesis in arcuate nucleus of DKO animals reduce *Gnrh1* expression and pulsatile GnRH secretion. This in turn reduces the expression of the gonadotroph-specific genes *Lhb, Fshb,* and *Gnrhr* in females and males. While *Gnrhr* gene expression is reduced, enough GnRHRs remain to trigger calcium signaling and LH secretion, as well spermatogenesis and oogenesis. However, reduced kisspeptin synthesis in the RP3V region affects the GnRH surge release required for ovulation and postovulatory steroidogenesis, leading to female infertility. Further studies are needed to confirm these hypotheses. Finally, the expression of *Ptprn* and *Ptprn2* in other secretory pituitary cells and their regulatory neurons in the hypothalamus raises the interest for the role of these genes in their functions.

## Methods

### Animals

All experiments with mice described in this study were done in accordance with the National Institutes of Health Policy Manual 3040-2: Animal Care and Use in the Intramural Program and were approved by the NICHD, Animal Care and Use Committee (Animal Protocol 19-041). Authors complied with the ARRIVE guidelines. DKO females and males were generated using *Ptprn*^*-/-*^ + *Ptprn2*^+*/-*^ as parents. Mice were genotyped using tail clips and DNA extraction and PCR were performed using the KAPA HotStart mouse genotyping kit (Kapa Biosystems, Sigma-Aldrich, St. Louis MO) in the presence of 0.5 µM specific primers. PCR products were separated by agarose gel electrophoresis (1.5% agarose; Sibr Safe DNA Gel Stain (1:66.7000) (ThermoFisher Scientific, MA), and product visualization was performed by FluorChem E (ProteinSimple, San Jose, CA). After genotyping at age of 21 days, animals were removed from parents’ cages and housed in groups of up to five littermates per cage. Animals were housed under conditions of constant temperature and humidity, with lights on between 6 AM and 8 PM. Experimental procedures were conducted on 90-150-day-old females and males. To determine the stage of the estrous cycle, vaginal lavage and smears were performed every morning for two weeks. Dry smears were stained with 0.1% methylene blue solution (Sigma-Aldrich, St. Louis MO) for 1 h, washed and analyzed. The precise stage of the estrous cycle, named proestrus, estrus, metestrus, and diestrus, was determined based on the presence/absence and abundance of live/dead epithelial cells and leukocytes as described^51,52^. Vaginal opening was used as an external index of puberty onset and the animals were monitored from the 24^th^ postnatal day as described^51,52^. Mice were euthanized by isoflurane (Baxter, Deerfield, IL) overdose, followed by decapitation. After decapitation blood was collected for hormonal analysis and hypothalamic tissue, pituitary glands, ovaries, and testes were removed, and used for cell preparation, qRT-PCR analysis, ELISA measurements, and/or Western blot analysis.

### Pituitary cell dissociation

Five WT and DKO mice were used for pituitary cell dissociation from both sexes. Pituitaries were collected in 10 mL HEPES-M199 medium (Gibco / Thermo Fisher Scientific, Waltham, MA), containing 10% heat inactivated horse serum (HS) and penicillin/streptomycin 1X from Gibco. After 2 rinses in 1.26 mM Ca^2+^-containing PBS (Gibco), supplemented with 3% bovine serum albumin (MP Biomedicals, Irvine, CA), penicillin/streptomycin 1X (Gibco), MEM vitamin 1X (Gibco), and 2 mM glutamine (Gibco), pituitaries were minced twice with a Mcllwain tissue chopper. Tissue fragments were then rinsed twice with Ca^2+^-containing PBS and incubated in Ca^2+^-containing PBS supplemented with 0.4% trypsin (Sigma) for ~ 15 min (37 °C, 45 rpm). DNase (Millipore/Sigma, Burlington, MA) was added for a brief incubation then the digestion was stopped by replacing medium with Ca^2+^-containing PBS supplemented with 2 mg/mL trypsin inhibitor (Millipore/Sigma). Fragments were transferred and flicked twice into Ca^2+^-deficient PBS supplemented with 2 mM EGTA, then supplemented with 1 mM EGTA, and finally moved in Ca^2+^-deficient PBS supplemented only with DNase. Fragments were progressively dissociated mechanically by pipetting up and down, supernatant collected, spined down then resuspended in sodium bicarbonate containing M199 (Gibco). For calcium imaging, cells were seeded in Poly-L-lysine-coated coverslips (0.01% w/v; Millipore/Sigma) and placed at 37 °C in a 5% CO_2_ humidified incubator to adhere. After 30 min, the coverslips were submerged in sodium bicarbonate-M199 supplemented with 10% heat inactivated HS and penicillin/streptomycin 1X, and imaged 2 days later. For secretory studies, cells were cultured in 24 well plates, 125,000 cells/well. At the end of incubation, the medium was collected, the cells were scraped in the presence of 1xPBS enriched with protease (Roche GmbH, Germany) and phosphatase inhibitors (Sigma-Aldrich, St. Louis, MO) and samples were uniformed based on protein levels determined by Pierce BCA protein assay kit (ThermoFisher Scientific, Waltham, MA).

### Immunohistochemistry

For GnRH and kisspeptin immunohistochemistry, adult WT and DKO mice (6 females and 3–4 males for each genotype), anesthetized with 2.5–3% isoflurane, were transcardially perfused with 0.1 M PBS, then 4% formaldehyde in PBS. The heads were postfixed (overnight). After postfixation, brains were removed and transferred to a 30% sucrose-PBS solution until the tissue sank. Brains were frozen in dry ice vertically on a drop of Tissue-Plus OCT (Fisher Healthcare/Thermo Fisher Scientific) and kept at − 80 °C until sectioning. The brains were cut into four sets of coronal Sects. (40 µm) with a Leica SM2010 R sliding microtome (Wetzlar, Germany) and kept at − 20 °C in cryoprotectant until staining (> 5 days). One set per animal was used for GnRH immunohistochemistry. After washes in PBS, free-floating sections were treated with a universal antigen retrieval solution (Abcam, Waltham, MA) for 30 min at 80 °C and washed in PBS. Then, endogenous peroxidase was blocked with PBS supplemented with 20% methanol, 0.2% Triton X-100, 3% H2O2 (15 min at room temperature). After PBS washes, free-floating sections were incubated for 1 h at room temperature in a blocking solution (10% normal horse serum plus 0.3% Triton X-100), washed several times in PBS, and incubated (2–3 nights, 4 °C) in primary antibody against GnRH (Immunostar, Hudson, WI; 1:15,000) or kisspeptin (Alain Caraty 564, Nouzilly, France; 1:5000). Sections were then washed in PBS, incubated (1 h, room temperature) with biotinylated secondary donkey anti-rabbit antibody (1:500 in PBS/0.3% Triton X-100; Vector Laboratories), washed in PBS, and processed for avidin–biotin horseradish peroxidase/3,3′-diaminobenzidine enhanced with nickel.

The sections were mounted, dried overnight and coverslipped with Permount mounting medium (Fisher Chemical/Thermo Fisher Scientific). The slides were randomly selected, and GnRH neurons were counted at 10X magnification. The GnRH neuron distribution was examined in sections from *organum vasculosum laminae terminalis* (cell body-rich area) and median eminence (fiber-rich area). The kisspeptin immunoreactivity was assessed along the RP3V and in the arcuate nucleus. Bright field images were taken through 10X objective (NA 0.3, WD 15.2) on Eclipse Ti2 Nikon microscope (Melville, NY) and captured with ORCA-Flash4.0 V3 digital camera (Hamamatsu, Bridgewater, NJ). The GnRH fiber density was assessed using FIJI^53^ at three levels of the tuberoinfundibular region (Approximate Bregma AP coordinates: − 1.94, − 2.06 and − 2.18, corresponding to levels L1, L2, and L3 in Fig. 8). Images were first binarized using the unstained tissue as threshold value. A constant circular region of interest was then positioned on the tuberoinfundibular tracts, and the staining density was defined as the percentage of pixels with a value above threshold within the region of interest. Similar method was used to quantify kisspeptin immunoreactivity in the RP3V and the arcuate nuclei.

Testicles collected from control and DKO mice were fixed in Bouin’s solution and embedded in paraffin. Sections, 7 μm thick, were cut on a microtome and routinely stained for hematoxylin after deparaffinization. Stained sections were observed under Leica DMRB microscope equipped with camera (Leica Microsystems GmbH, Wetzlar, Germany). Micrographs were sized, cropped, and arranged in Photoshop CS (Adobe Inc. San Jose, CA).

### qRT-PCR analysis

Total hypothalamic RNA was isolated by Trizol reagent (300 µl/hypothalamus; Ambion, Austin, TX) with addition of chloroform (60 µl/sample). After shacking and centrifugation (12000xg/15 min/4 °C) supernatant was collected, RNA was extracted by isopropyl alcohol (250 µl/sample) and centrifugation (12,000×*g*/10 min/4 °C). Collected RNA was washed twice by 75% ethanol, resuspended in RNase-free water and genomic DNA was eliminated by gDNA Eliminator (RNeasy Plus, Qiagen, Valencia, CA) (Tamar et al. 2005). Total RNA from pituitary, ovarian, and testicular tissues was extracted using RNeasy Plus Mini Kit (Qiagen, Valencia, CA) and reverse transcribed with the Transcriptor First Strand cDNA Synthesis Kit (Roche Applied Sciences, Indianapolis, IN). qRT-PCR was performed using pre-designed Taq-Man Gene Expression Assays for mice, TaqMan fast advanced master mix and QuantStudio 6 Felx RT-PCR system (Applied Biosystems, Waltham, MA). Target gene expression levels were determined by the comparative 2^-(delta C_t_) quantification method using *Gapdh* as the reference gene, which was previously established to be a suitable for neuroendocrine cells^26^. Applied Biosystems predesigned TaqMan Gene Expression Assays were used: *Ptprn* (Mm01258989_m1), *Ptprn2* (Mm01229147_m1), *Cga* (Mm01209400_m1), *Cyp11a1* (Mm00490735_m1), *Cyp17a1* (Mm00484040_m1) *Cyp19a1* (Mm00484049_m1), *F*s*hb* (Mm00433361_m1), *Fshr* (Mm00442819_m1), *Gapdh* (Mm99999915_g1), *Gnrh*1 (Mm01315604_m1), *Gnrhr* (Mm00439143_m1), *Hsd3b1* (Mm00476184_g1), *Hsd17b1* (Mm00501692_g1), *Kiss1* (Mm03058560_m1), *Kiss1r* (Mm00475046_m1), *Lhb* (Mm01205505_g1), *Lhr* (Mm00442931_m1), *Nr5a1* (Mm00446826_m1), *Pdyn* (Mm00457573_m1), *Spp1* (Mm00436767_m1), *Star* (Mm00441558_m1), and *Tac2* (Mm01160362_m1).

### Protein isolation and western blot

Proteins were isolated from homogenized individual mouse pituitaries using RIPA buffer (Sigma-Aldrich, St. Louis, MO) enriched with protease (Roche GmbH, Germany) and phosphatase inhibitors (Sigma-Aldrich, St. Louis, MO) and uniformed using Pierce BCA Protein Assay Kit (ThermoFisher Scientific, Waltham, MA). Samples (20 µg of proteins in each lane) were loaded onto 4–15% Mini-PROTEAN TGX precast SDS- polyacrylamide gels, separated by electrophoresis using Mini-PROTEAN tetra cell system and transferred onto 0.2 μm PVDF membranes using Trans-Blot Turbo Transfer System (all obtained from Bio-Rad, Hercules, CA). Membranes were blocked for 2 h at room temperature with 5% nonfat-dried milk (Bio-Rad) in TBST (0.5 M Tris Base, 9% NaCl, 1.5% Tween 20) and then incubated with anti-LHB antibody (1:1000) overnight at 4 ℃. The next day, membranes were incubated with horseradish peroxidase conjugated secondary anti-rabbit antibody (1:5000) for 1 h at room temperature. Membranes were incubated with anti-GAPDH antibody (1:1000) overnight at 4 ℃, followed by incubation with horseradish peroxidase conjugated secondary anti-mouse antibody (1:5000) for 1 h at room temperature. Anti-LHB antibody was obtained from Dr. A. F. Parlow (National Institute of Diabetes and Digestive and Kidney Diseases, National Hormone and Peptide Program, Torrance, CA) and GAPDH and both secondary antibodies were purchased from Santa Cruz (Dallas, TX). All antibodies were diluted in TBST containing 5% nonfat-dried milk. After each step, membranes were washed three times in TBST. Chemiluminescent signal was induced by SuperSignal West Femto Maximum Sensitivity Substrate and visualized using the iBright CL1500 Imaging System (both from Thermo Fisher Scientific). Quantification was done by Adobe Photoshop (Adobe, San Jose, CA).

### Calcium imaging

HEPES-buffered Krebs–Ringer (KR) solution (in mM: NaCl 145, KCl 4.5, CaCl_2_ 2, MgCl_2_ 1, HEPES 10, Glucose 10) was made from a 10X stock solution the morning of the experiment. Drugs were diluted from 1000X-10000X aliquots immediately prior to experiments. Fura-2 AM was dissolved in 1 mM DMSO and diluted down to 2 µM in KR solution. Pituitary cells were incubated for 1 h at room temperature in Fura-2 containing KR. After a rinse in KR solution, coverslips were mounted into a Warner Instrument chamber/platform combination (RC-21BRW/P2; Holliston, MA) adapted to an inverted Eclipse Ti2 Nikon microscope. Pituitary cells were visualized through an oil-immersed 40X objective (NA 1.3, WD 0.24) and imaged at room temperature. Perfusion was set at a flow rate of ~ 1.4 mL/min and solutions switched with a gravity-fed solution changer RSC-200 (BioLogic Science Instruments, Knoxville, TN). Excitation bandpasses at 348/30 nm and 380/20 nm were provided by a Retra II light engine (Lumencor, Beaverton, OR), and emission bandpass was detected through a 510/84 nm filter. Images were acquired every second with an ORCA-Flash4.0 V3 digital camera (Hamamatsu, Bridgewater, NJ). Both illumination and image acquisition were piloted by NIS-Elements (Nikon). Optical densities of background and cells at both excitation wavelengths over time were measured post-hoc with NIS-Elements and data imported into Matlab for processing (MathWorks, Natick, MA). Background values at 340 and 380 nm were subtracted from each cell value at their respective excitation wavelengths for each time point then the 340/380 nm ratio was calculated and plotted. To determine the percentage of gonadotrophs per coverslip, images at the GnRH application were binarized, thresholded, and the area seeded with cells was measured as pixels above threshold. Cells were then counted manually on 6 coverslips and the average size of a single cell was estimated (pixels/cell). The number of cells was then estimated by divided the area seeded by the average size of a single cell. The percentage of gonadotrophs was the ratio of GnRH-responsive cells and estimated cell numbers. Gonadotroph data were analyzed using a custom-made macro in Matlab (Natick, MA). Gonadotrophs were identified as cells that displayed a change in calcium levels (optical density, OD) greater than 20% between 3 min before GnRH and 3 min after GnRH. Calcium oscillations in the response to GnRH was determined as dOD/dt greater than 0.07 in the presence of GnRH. The time between calcium oscillations was calculated and the frequency of calcium oscillations was expressed as the number of calcium oscillations per 1 s. The area under the curve (AUC) was determined as the sum of OD from the onset of the response until the removal of GnRH, relative to baseline calcium level defined 3 min before GnRH.

### Hormone assays

Testosterone and progesterone in serum and gonads were determined using ELISA kits of the Cayman Chemical (Ann Arbor, MI), following their instructions. Pituitary LH content and concentration were measured using an ELISA kit from Endocrine Technologies (Newark, CA) and measurements were done according to the manufacturers’ instructions. To determine gonadal steroids testes and ovaries were homogenized with 1xPBS or 0.2%EDTA-1xPBS (V: W = 10 µl: mg) respectively. Steroids were extracted from the homogenate (50–100 µl) by ethyl ether, resuspended in the assay buffer and determined following manual instructions (Cayman Chemical) with minor modifications.

### Statistical analysis

Results are presented as mean ± SEM values, with number of replicates indicated in figures. Student *t* test was used to determine if there is a significant difference between the means of two groups. Two-way ANOVA and subsequent Sidak’s multiple comparison test were used to conduct the test with three or more means. P < 0.01 was considered as statistically significant. Mean ± SEM values and statistical analysis were calculated, and initial graphs generated using the KaleidaGraph program (Synergy Software, Reading, PA). Figures were finalized using Adobe Illustrator and Photoshop.

## Supplementary Information

Supplementary Figures.

## Data Availability

All relevant data are within the paper.

## Abbreviations

FSH: Follicle-stimulating hormone
FSHR: FSH receptor
DKO: Knockout of *Ptprn* + *Ptprn2* genes
GnRH: Gonadotropin-releasing hormone
LH: Luteinizing hormone
LHB: Luteinizing hormone subunit beta
LHR: Luteinizing hormone/chorionic gonadotropin receptor
PTPRN and PTPRN2: Protein tyrosine phosphatase receptor type N and type N2
RP3V: Rostral periventricular region of the third ventricle
WT: Wild type
