# Supplementary material for: Common and female-specific roles of protein tyrosine phosphatase receptors N and N2 in mice reproduction
Source: Sci Rep. 2023 Jan 7;13:355. doi: 10.1038/s41598-023-27497-4 (PMC9825377; doi:10.1038/s41598-023-27497-4)
Supplement: Supplementary file 1 — Supplementary Figures. [file 41598_2023_27497_MOESM1_ESM.docx]

**Supplementary Material**

Common and Female-Specific Roles of Protein Tyrosine Phosphatase Receptors N and N2 in Mice Reproduction

Srdjan J. Sokanovic^1^, Stephanie Constantin^1*^, Aloa Lamarca Dams^1^, Yuta Mochimaru^1^, Kosara Smiljanic^1^, Ivana Bjelobaba^2^, Rafael M. Prévide ^1^, Stanko S. Stojilkovic^1^

^1^Section on Cellular Signaling, The Eunice Kennedy Shriver National Institute of Child Health and Human Development, National Institutes of Health, Bethesda, MD 20892, USA, ^2^Department for Neurobiology, Institute for Biological Research “Siniša Stanković” - National Institute of Republic of Serbia, University of Belgrade, Bulevar despota Stefana 142, 11000, Belgrade, Serbia.

**Figure S1.** Expression of *Ptprn* and *Ptprn2* genes in gonads, pituitary gland, and hypothalamic tissues from wild type (WT) male and female mice. Data points are mean ± SEM values, with number of replicates 10 per group.


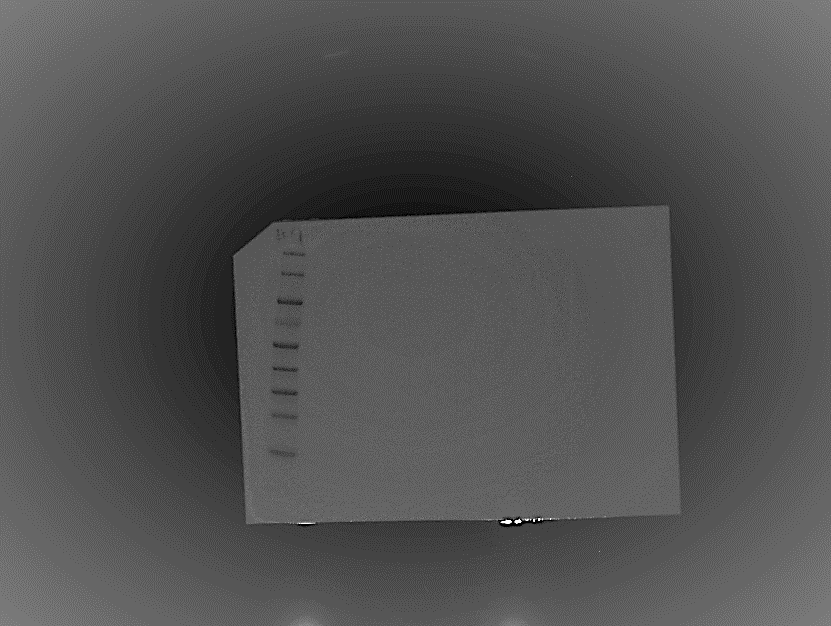

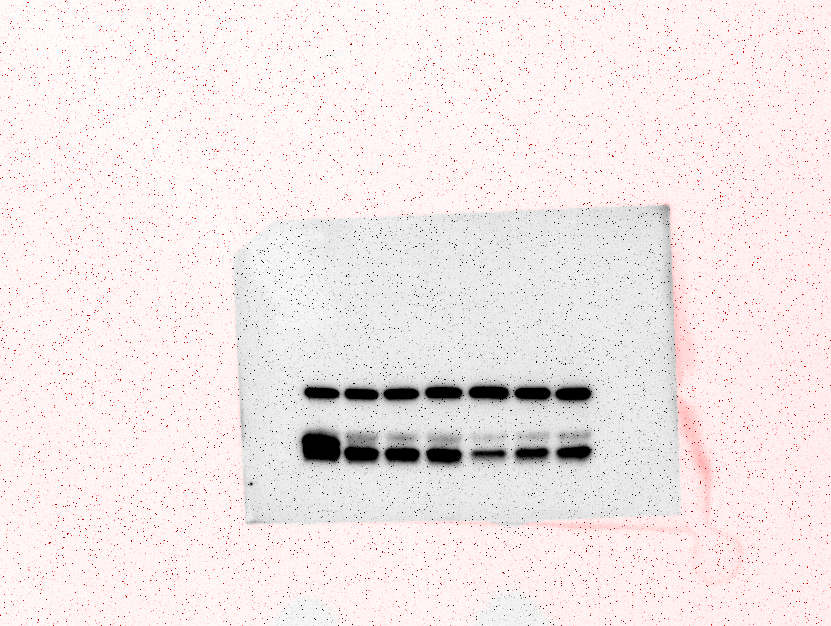

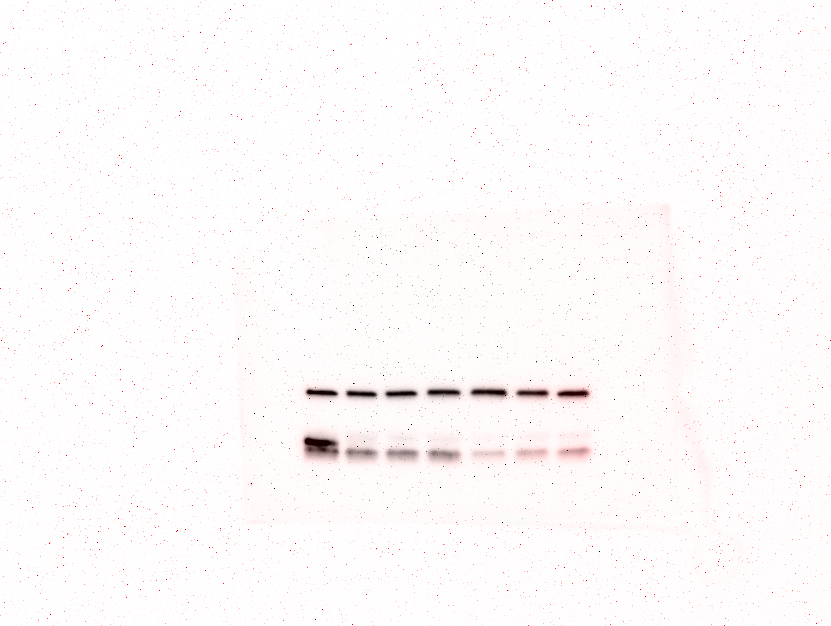

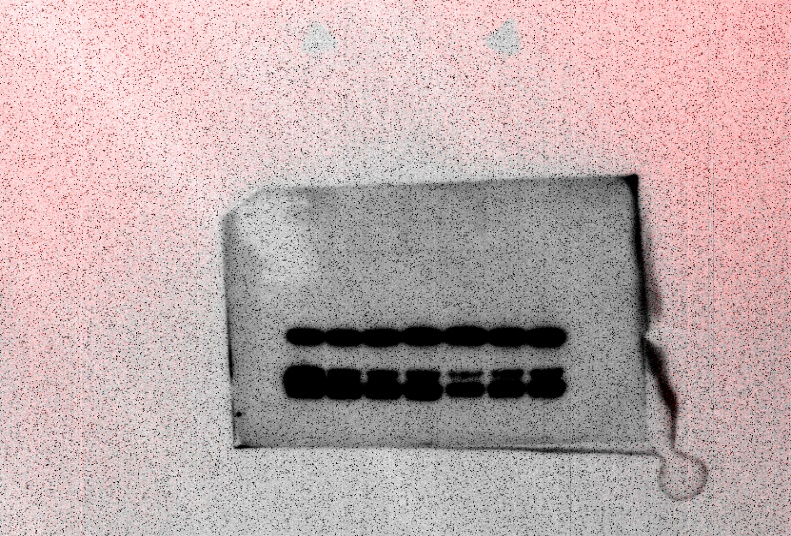


**15kDa**

**15kDa**

**15kDa**

**35kDa**

**15kDa**

**D**

**C**

**B**

**A**

**LHB**

**Figure S2.** Western blot analysis of LHB expression in male pituitaries from WT and DKO mice. (A-C) Multiple exposures of the original whole western blots are shown. The highlighted horizontal bands labeled LHB were cropped from image A by Photoshop. The first sample on the left was excluded from further analysis; all other samples were set up simultaneously, used for quantification, and plotted in Figure 4A, top panel. (D) The weight of the bands was determined based on the protein ladder.


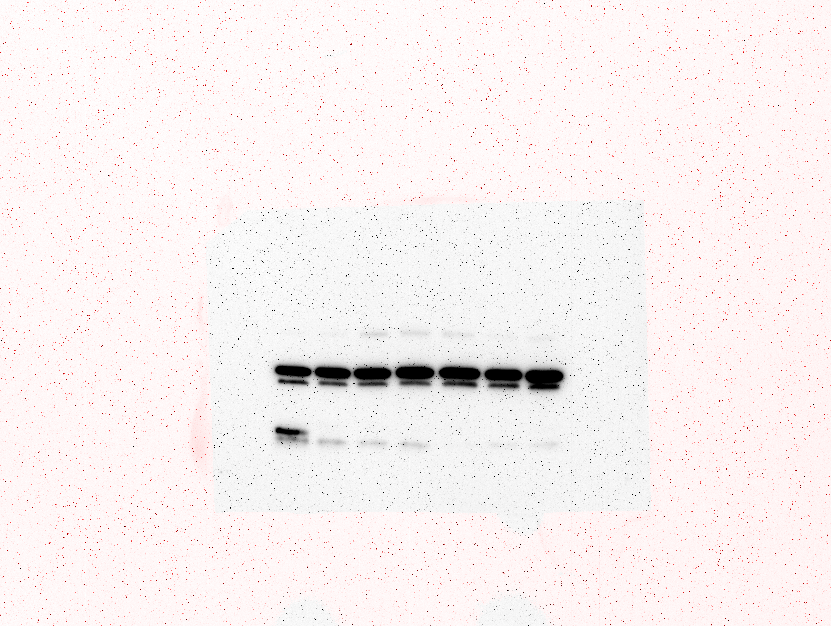


**GAPDH**

**35kDa**

**35kDa**

**15kDa**

**15kDa**

**GAPDH**

**B**

**A**


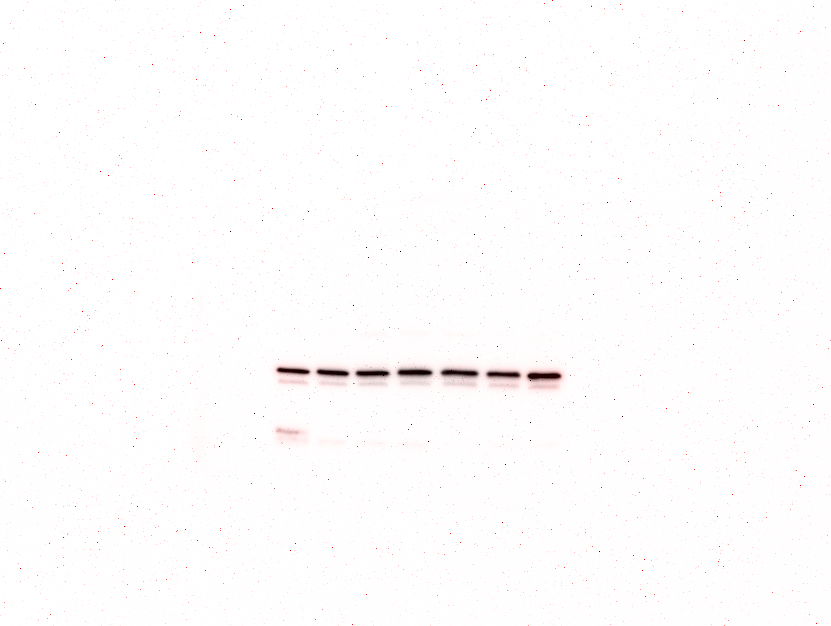


**D**

**C**


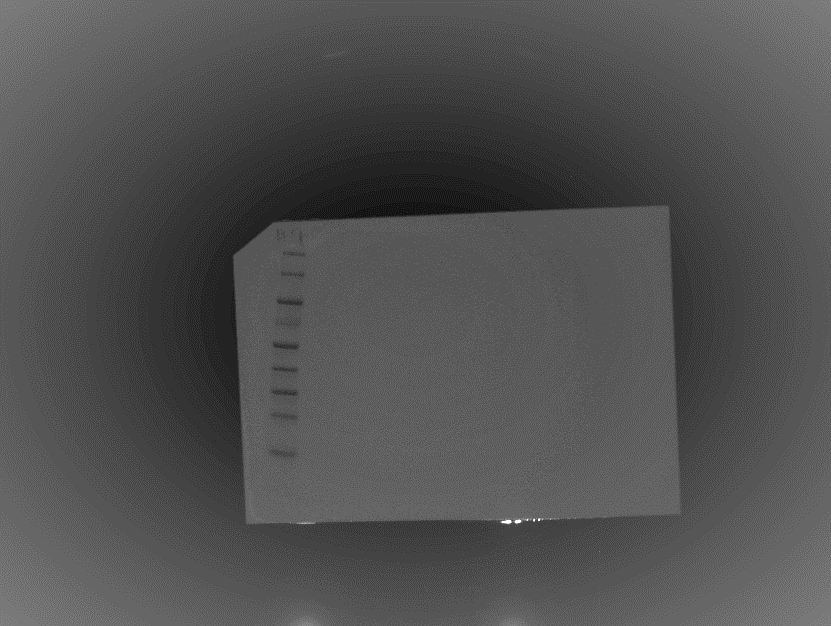

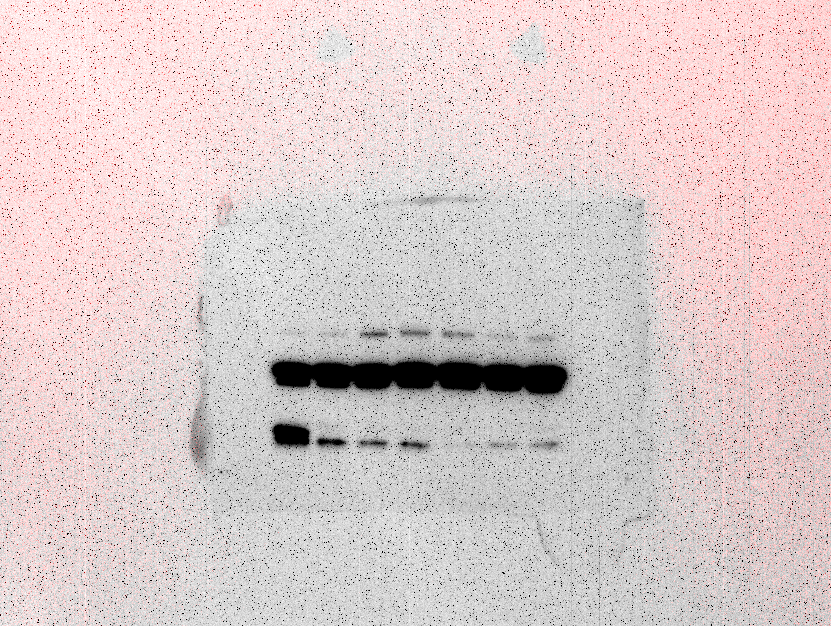


**35kDa**

**15kDa**

**35kDa**

**15kDa**

**GAPDH**

**Figure S3.** Western blot analysis of GAPDH expression in male pituitaries from WT and DKO mice. (A-C) Multiple exposures of the original whole western blot are shown. The outlined horizontal bands labeled GAPDH were cropped from image A by Photoshop. The first band was excluded from further analysis; all other samples were set up at the same time, used for quantification, and plotted in Figure 4A, top panel. (D) The weight of the bands was determined based on the protein ladder.

**A**

**B**


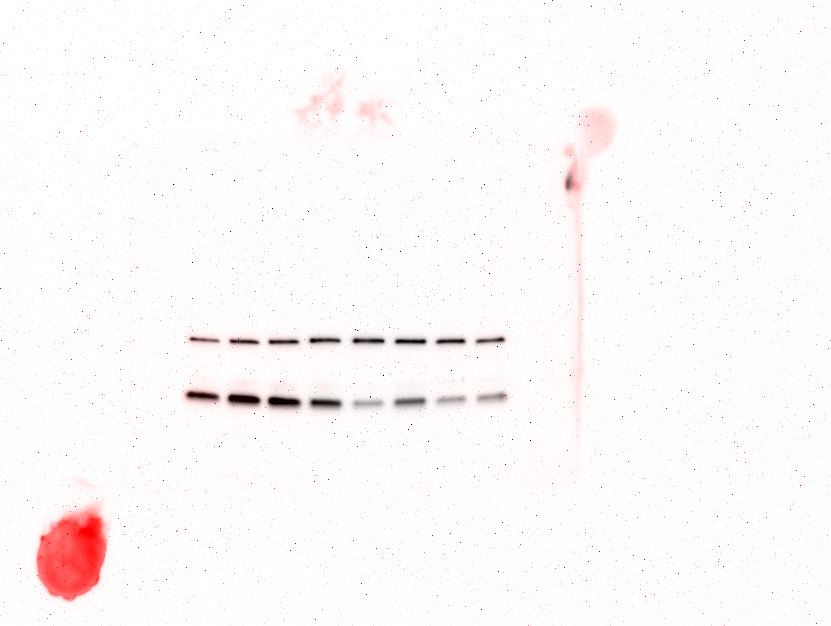

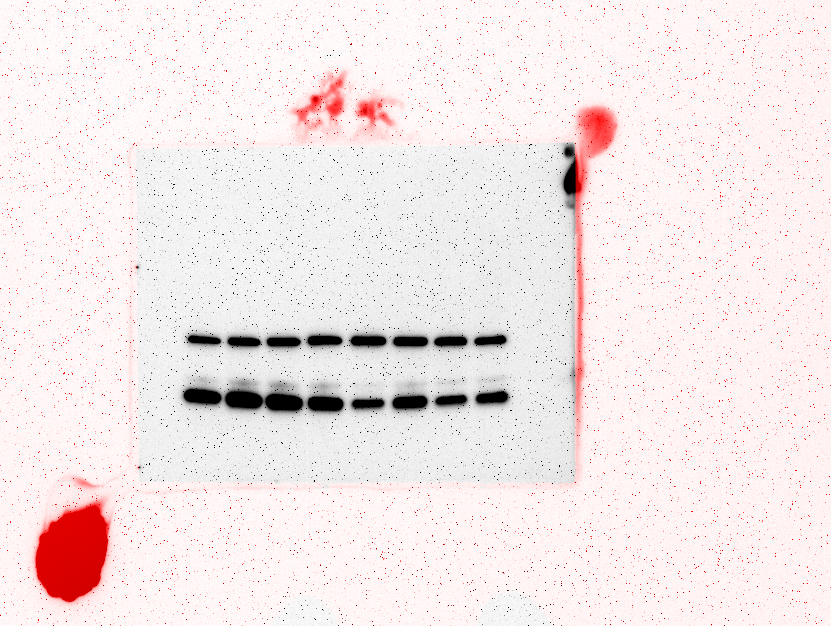


**15kDa**

**15kDa**

**LHB**

**LHB**


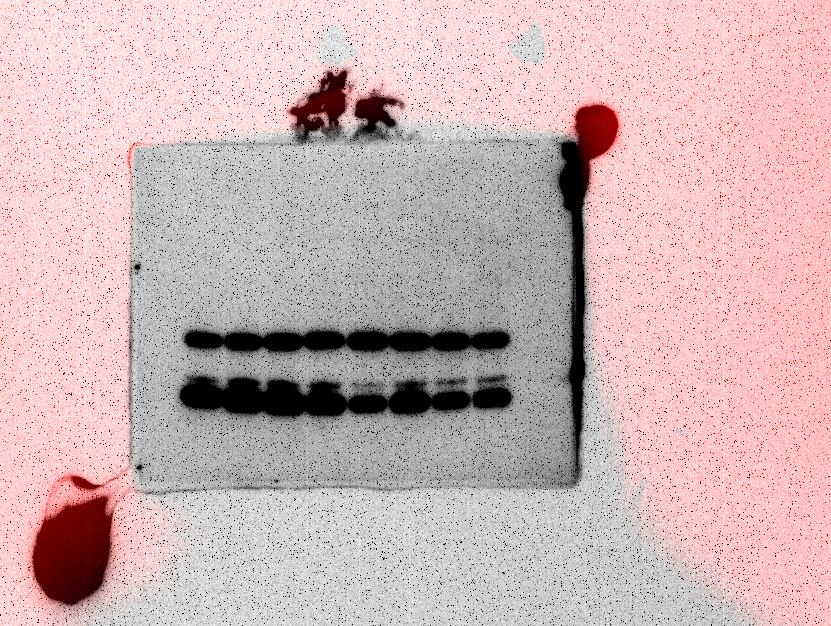

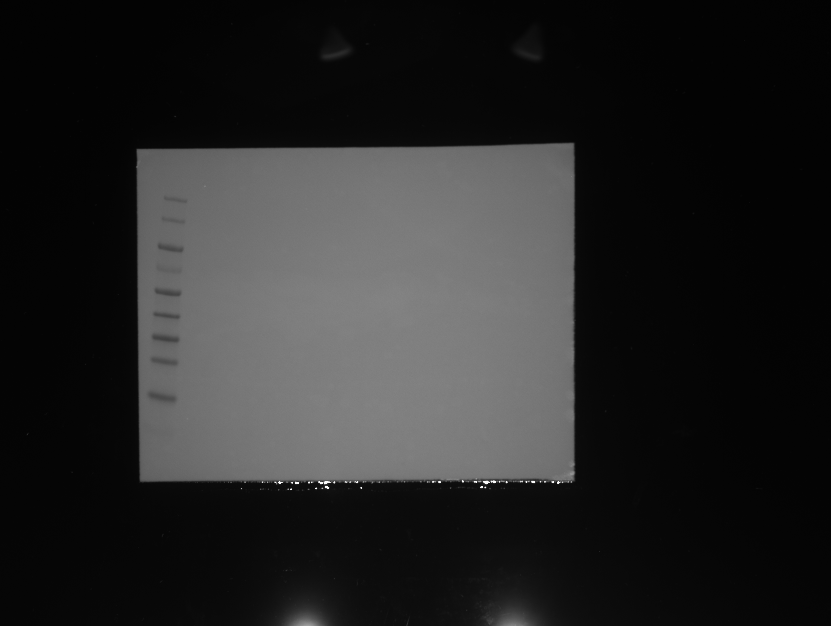


**15kDa**

**15kDa**

**LHB**

**C**

**D**

**35kDa**

**Figure S4.** Western blot analysis of LHB expression in female pituitaries from WT and DKO mice. (A-C) Multiple exposures of the original whole western blots are shown. The outlined horizontal bands labeled LHB were cropped from image A by Photoshop. All samples were set up simultaneously, used for quantification, and plotted in Figure 4A, bottom panel. (D) The weight of the bands was determined based on the protein ladder.

**B**

**A**


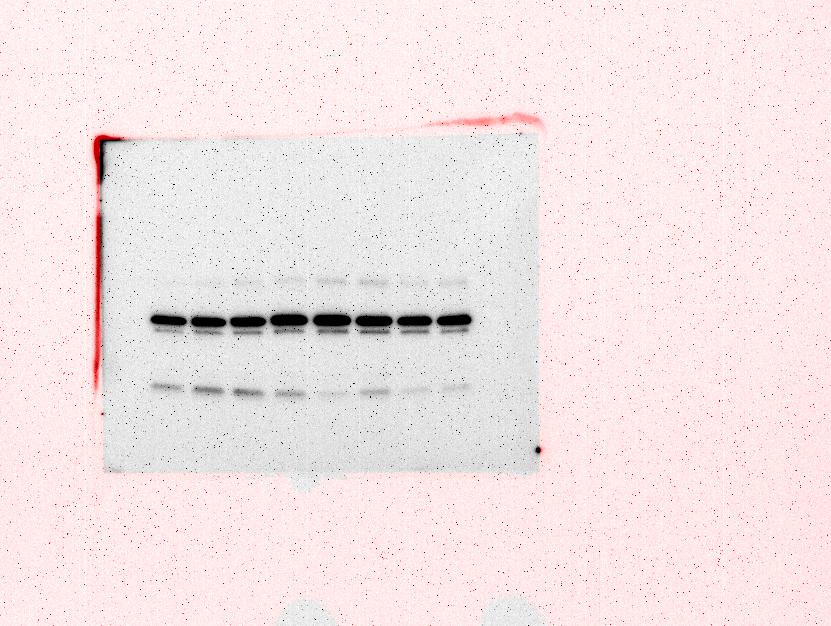


**15kDa**

**15kDa**

**35kDa**

**35kDa**

**GAPDH**

**GAPDH**


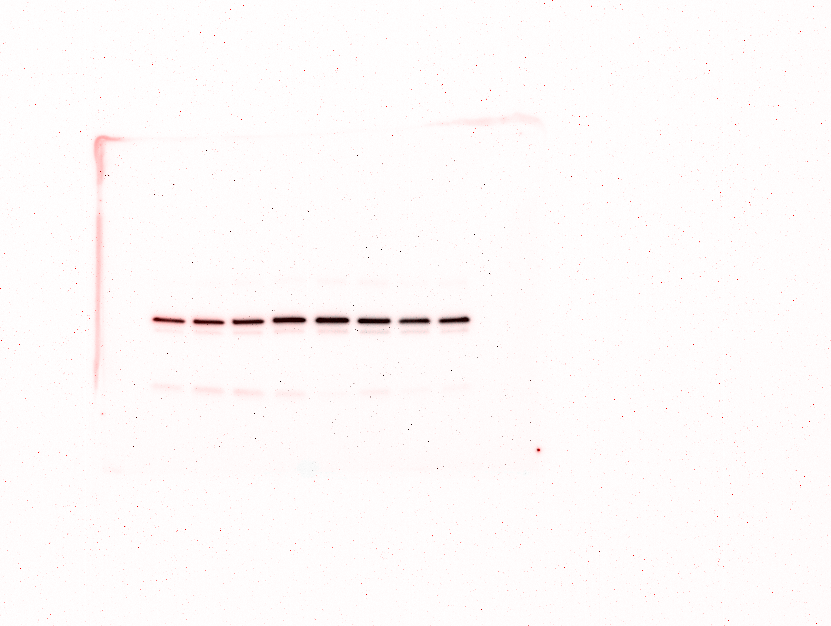


**C**

**D**


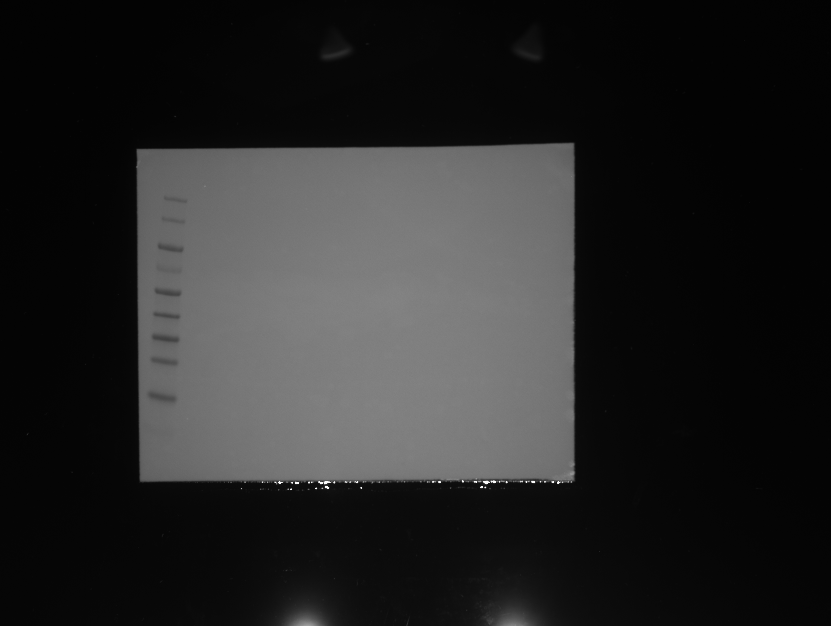

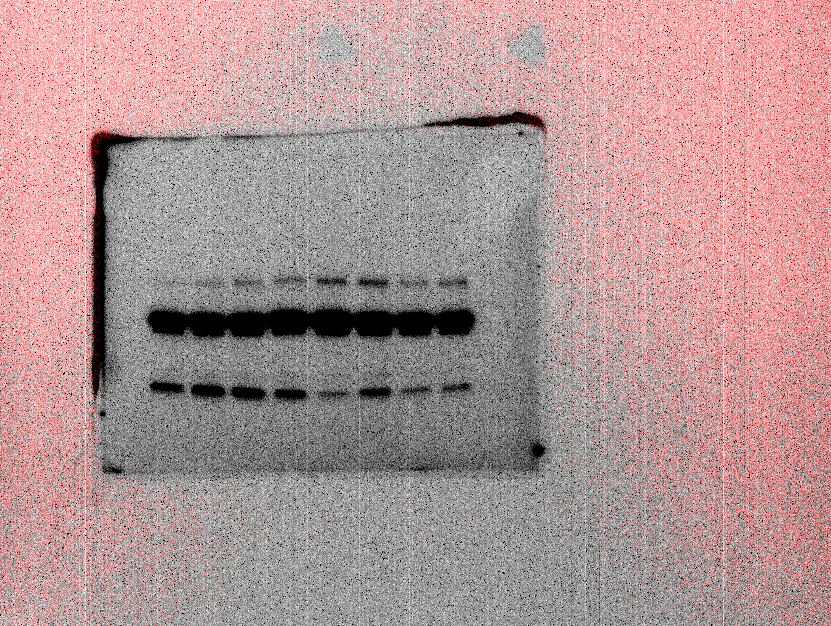


**35kDa**

**15kDa**

**35kDa**

**15kDa**

**GAPDH**

**Figure S5.** Western blot analysis of GAPDH expression in female pituitaries from WT and DKO mice. (A-C) Multiple exposures of the original whole western blot are shown. The outlined horizontal bands labeled GAPDH were cropped from image A by Photoshop. All samples were set up at the same time, used for quantification, and plotted in Figure 4A, bottom panel. (D) The weight of the bands was determined based on the protein ladder.
